# Deep Eutectic Solvents as a Sustainable Approach for Silica Recovery from Rice Husk

**DOI:** 10.3390/molecules30244697

**Published:** 2025-12-08

**Authors:** Célio S. Faria-Júnior, Lucas dos Santos Silva, Armando L. C. Cunha, Filipe S. Buarque, Bernardo Dias Ribeiro

**Affiliations:** School of Chemistry, Federal University of Rio de Janeiro, Av. Athos da Silveira Ramos, 149. Ilha do Fundão, Rio de Janeiro 21941-909, Brazil; celio-junior28@eq.ufrj.br (C.S.F.-J.); lucasds12silva@gmail.com (L.d.S.S.); armando@eq.ufrj.br (A.L.C.C.); bernardo@eq.ufrj.br (B.D.R.)

**Keywords:** green chemistry, DES, lignocellulosic biomass, rice husk, biosilica

## Abstract

Rice husk is a lignocellulosic biomass rich in silica, which, when disposed of inappropriately, represents an environmental hazard. This study investigated the application of deep eutectic solvents (DES) as a green and efficient approach to the rice husk fractionation, combining the selective dissolution of lignin and sugars with the purification of the silica-rich inorganic fraction. Six different DES were produced from choline chloride or betaine with different hydrogen bond donors and characterized for water content and pH. The DES based on carboxylic acids was more acidic, which favored the cleavage of ester and glycosidic bonds in the biomass. The TGA, XRF, SEM, and XRD analyses revealed that the lactic acid-based DES promoted better removal of lignin and mineral impurities, resulting in a purer silica with an amorphous morphology. The 110 °C condition was the most effective in preserving the thermal integrity of the organic (sugars and lignin) and inorganic (silica-rich ash) fractions. The results highlight the potential of DES as selective, sustainable, and tunable solvents for the valorization of agricultural waste, achieving biosilica with SiO_2_ purity exceeding 80% and lignin removal above 70%, reinforcing the potential of DES as sustainable solvents for agricultural waste valorization.

## 1. Introduction

The agro-industrial sector plays a crucial role in a nation’s socioeconomic development, responsible for producing food, fiber, and bioenergy [1]. With the increasing adoption of the circular economy concept, this sector has evolved toward a more sustainable and efficient production model [2]. The valorization of agro-industrial waste as an alternative source of high-value-added products is a promising strategy for reducing environmental impacts and increasing industrial competitiveness, aligning with the Green Chemistry guidelines [3,4].

Rice (*Oryza sativa*) is a staple food consumed globally and holds a vital position in the international agro-industrial market. It is the second most consumed cereal and generates valuable by-products that can be utilized within the circular economic framework [5]. In 2023–2024, global rice production is projected to range between 513.5 and 523.9 million metric tons [6]. Brazil, the world’s ninth-largest producer and exporter of this grain, is expected to produce approximately 10.8 million metric tons, an increase from the previous year [7]. About 20% of the rice grain’s weight consists of the husk, an abundant and low-cost agro-industrial by-product [8]. However, a significant portion of rice waste, particularly the husk, remains unutilized, leading to considerable environmental impacts. Therefore, the efficient processing of rice husks can provide economic and environmental benefits by converting them into value-added materials [9,10].

This residue possesses properties that make it suitable as a raw material for bioethanol production, as its composition includes cellulose, hemicelluloses, and lignin, common characteristics of lignocellulosic biomass [10]. Additionally, rice husks can be utilized within the rice industry itself as an energy source for boilers, dryers, and autoclaves due to their high calorific value, reaching temperatures of up to 1000 °C (approximately 16,720 kJ kg^−1^), equivalent to 33% of the thermal capacity of oil. However, the high temperatures used in this process degrade the organic matter in the husk, resulting in the formation of ash, which constitutes its inorganic fraction (15–28%). This ash primarily comprises 87–97% silica, a highly porous, lightweight material with a large surface area, making it highly valuable for industrial applications [11,12].

Silica (SiO_2_) is a naturally occurring mineral that is water-insoluble and widely utilized in glassware, polymer, ceramics, and construction industries due to its unique properties. Additionally, silica serves as a primary raw material for the production of various organic and inorganic compounds used as catalysts in chemical processes, as well as in electronic and optical coatings [13,14]. However, extracting silica from rice husks presents several challenges. Conventional methods involve burning the husk to obtain ash, but improper combustion can release atmospheric pollutants and reduce silica reactivity due to particle sintering. Alternatively, chemical extraction using acids or bases can generate liquid waste with significant environmental impacts [15,16].

To address these challenges, innovative silica extraction methods aligned with Green Chemistry principles have been explored to minimize hazardous waste generation and energy consumption [17]. One such approach involves the use of deep eutectic solvents (DES), first described by Abbott et al. [18] as liquid mixtures composed of two or more components, including a hydrogen bond acceptor (HBA) and a hydrogen bond donor (HBD). These components form intermolecular associations through hydrogen bonds, lowering the system’s melting point compared to its pure constituents due to charge delocalization facilitated by hydrogen bonding [19,20,21]. DES are considered design solvents due to their tunable physicochemical properties, which can be tailored for specific applications [22,23]. Recent studies have highlighted their potential in biomass delignification, particularly their ability to cleave β-O-4 bonds in lignin [24,25]. Alvarez-Vasco et al. [26] demonstrated that a mixture of choline chloride (ChCl) and lactic acid exhibits high selectivity in breaking ether bonds within the lignin structure, leading to the formation of Hibbert ketones, a mechanism analogous to conventional acidolysis. Furthermore, recent research suggests that the halide anion in ChCl can function as a nucleophile, facilitating the substitution of hydroxyl groups adjacent to ether bonds. This interaction promotes the formation of stable intermediates, ultimately enhancing lignin degradation efficiency.

Given this context, the main objective of this work was to investigate the application of DES for the fractionation of rice husks, combining the selective solubilization of the organic fraction (lignin and sugars) with the recovery and purification of the silica-rich inorganic fraction. For this purpose, the efficiency of different DES in solubilizing lignin and sugars was evaluated, while the treated biomass and the resulting biosilica were characterized using physicochemical techniques (TGA, XRF, SEM, and XRD), and the process operating conditions, such as temperature and solvent composition, were optimized.

## 2. Results and Discussion

The DES characterization (water content, pH, and thermal stability by TGA) is displayed in the Supporting File, Tables S1 and S2.

### 2.1. Characterization of Rice Husks

#### 2.1.1. Physico-Chemical Characterization

The chemical composition of rice husk limits its direct use in food applications, and it is predominantly utilized in industrial processes. Although rich in fiber, its high silica and lignin contents restrict its incorporation into animal feed, as these substances can cause abrasion in the gastrointestinal tract [7]. As expected, low concentrations of proteins (1.95%) and lipids (0.79%) were quantified in raw rice husk, values consistent with those reported in the literature [27]. The carbohydrates present in rice husk are primarily composed of cellulose, hemicellulose, and lignin, which are essential components of the lignocellulosic structure. The indirect quantification of these compounds can be performed using the following formula:

Carbohydrates (%) = 100% − (lipids (%) + proteins (%) + ash (%) + moisture (%))(1)

Regarding the inorganic components, in natura rice husks have a low moisture content (7.52%), a feature that favors their application in the production of biofuels and in chemical processes that require less interference from moisture, such as pyrolysis and carbonization [28,29]. This factor is crucial for the efficiency of amorphous silica extraction, which is directly affected by the moisture content of the biomass. The ash content of rice husks in natura is considerable, reaching values of around 18.61%, which is above the average reported in the literature, which varies between 14% and 18% [30,31]. This high ash content contributes to the low biodegradability of biomass, which generates environmental concern [32]. The silica present in the ash could be a viable solution to mitigate the environmental impacts associated with the accumulation of this waste, in addition to adding value to the by-product of the rice industry.

Table 1 shows the physicochemical characterization of rice husks before and after carbonization. The drastic increase in the ash content of the carbonized husk (91.36%) is evidence of the removal of the organic fraction, leaving predominantly silica and other minerals. These results are in agreement with the literature, in which Shen et al. [33] describes carbonization as an effective process for concentrating the silica in the residual biomass matrix. The impact of the chemical treatment on the composition of the rice husk is also evident in the significant reduction in the carbohydrate content, from 71.13% in the raw husk to just 5.18% in the carbonized husk. This phenomenon is associated with the thermal degradation of cellulose and hemicellulose, leading to greater exposure of silica and facilitating its extraction.

#### 2.1.2. Thermogravimetric Analysis (TGA)

**Figure 1:**
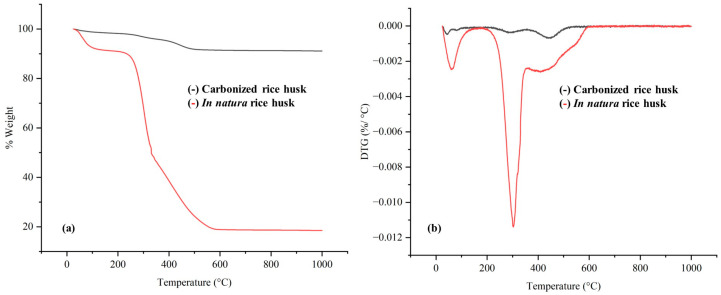
Thermogravimetric (**a**) and derivative thermogravimetric (DTG) (**b**) curves of raw and carbonized rice husks, illustrating mass loss profiles and thermal decomposition stages as a function of temperature.

TGA was used to characterize the rice husk, providing valuable information on its thermal stability and the decomposition processes associated with the biopolymers present. The analysis of the rice husk in natura revealed two significant mass losses in the temperature ranges of 25–120 °C and 300–450 °C. As shown in Figure 1 and Table 2, the first is related to the evaporation of residual moisture, while the second corresponds to the degradation of the organic components of the biomass. The decomposition process takes place in three main stages: hemicellulose, due to its amorphous structure, degrades between 200–260 °C; cellulose degrades between 240–260 °C; and lignin, due to its highly aromatic and polymerized structure, degrades more slowly and progressively, and can occur from lower temperatures to approximately 950 °C [34,35]. The higher thermal stability of lignin is attributed to its structural complexity and the presence of cross-links [36]. The TGA results corroborate these observations, showing that the rice husk has a total mass loss of approximately 59.92% up to 500 °C, indicating the conversion of the organic constituents. Nevertheless, the thermal decomposition of rice husk chemically treated with DES revealed a different thermal behavior. The interaction between the biopolymers and the solvents modified their thermal properties, possibly due to the formation of hydrogen bonds and alteration of the crystalline structure of the cellulose [35]. The treatment reduced the overlap of the hemicellulose and cellulose degradation bands, suggesting a structural modification caused by the extraction process.

On the other hand, the carbonized rice husk showed a TGA profile similar to that of the raw husk, but with significantly lower mass losses. This indicates that carbonization removes a large part of the organic material, leaving predominantly carbonaceous and mineral residues. However, the presence of small amounts of moisture and residual organic matter suggests that the carbonization process was not totally efficient, as evidenced by the detection of granules with a color characteristic of the bark in natura. In addition, TGA of the samples revealed that the carbonized material maintains greater thermal resistance, due to the high concentration of fixed carbon and silica in the final structure. Nguyen et al. [8] also found similar results when analyzing the thermal decomposition of rice husks. In their study, it was observed that hemicellulose degrades at lower temperatures, while cellulose degrades at a slightly higher range, and lignin shows a broader and more gradual decomposition. In addition, this study highlighted the influence of the purification of the lignocellulosic components on the thermal stability of the samples, which corroborates the findings of the present work. The similarity between the results reinforces the reliability of TGA analyses in characterizing rice husks and their treated derivatives.

#### 2.1.3. X-Ray Fluorescence (XRF)

Chemical analysis by XRF was used to determine the elemental composition in natura and carbonized rice husks, as shown in Table 3. The XRF technique is widely used due to its high sensitivity and precision in identifying the elements present in materials. The silica contents obtained were 76.36% for the carbonized rice husk and 27.6% for the raw husk, values which are in line with those as previously reported by Sathyamoorthi et al. [37]. The significant increase in silica concentration after carbonization is directly related to the elimination of the organic fraction of the biomass, reducing the total mass of the material and consequently increasing the proportion of mineral components. This enrichment of silica turns carbonized bark into a promising material for technological applications, such as the production of ceramic materials, catalysts, and high-performance compounds [38].

The results indicated that carbonized rice husk, without additional treatment, contains approximately 24% of other minerals, composed mainly of potassium, calcium, silver, iron, aluminum, and phosphorus. The presence of these elements can have a significant impact on the industrial application of the extracted silica, especially in processes that require high purity of the material, such as the manufacture of electronic devices and catalysts. These impurities can interfere with thermal stability, electrical conductivity, and surface chemistry of the silica, thus limiting its applicability. Furthermore, the high percentage of SiO_2_ observed in the carbonized sample highlights the efficiency of the thermal process in concentrating the mineral phase. However, to achieve silica purity levels greater than 95–99%, which are required for advanced materials (e.g., semiconductors), complementary chemical purification steps are necessary.

In addition, TGA of the carbonized rice husk showed that the thermal process promotes the gradual removal of organic compounds, resulting predominantly in mineral residues, especially silica. Studies such as that by Lee et al. [10] emphasize the relevance of using alternative solvents, such as ionic liquids and deep eutectic solvents, for removing metallic impurities. In this context, XRF provides a fundamental baseline for assessing the efficiency of both the thermal pre-treatment and any subsequent purification strategy. The reduction of undesirable oxides and the relative increase in SiO_2_ content serve as key indicators of process success in biomass valorization strategies.

Table 3 shows that treatment with the betaine: lactic acid system (1:2) led to a significant increase in the relative SiO_2_ content (from 76.36% in the untreated carbonized sample to 83.10% after treatment with this DES), which is evidence of its effectiveness in removing competing mineral impurities. Moreover, when using DES, there was a significant reduction in the concentrations of metal oxides such as K_2_O, CaO, and Ag_2_O, indicating that the process not only preserves the silica fraction, but also acts selectively in the elimination of alkali and alkaline earth metals. For example, the K_2_O content was reduced from 13.02% to 8.31% with the use of betaine: lactic acid, while CaO fell from 1.78% to 1.12% and Ag_2_O from 2.23% to 1.03%. These results are relevant since the presence of potassium and calcium can compromise the thermal and structural properties of silica.

Among the different DES evaluated, solvents based on lactic and oxalic acid showed the best performance in purifying silica, probably due to their moderate acidity and complexing capacity, which favor the solubilization of metal ions without degrading the inorganic matrix. On the other hand, DES with urea showed lower selectivity, which is reflected in higher levels of residual impurities. The data reinforce the hypothesis that DES act through selective extraction mechanisms mediated by acid-base interactions and the formation of soluble complexes with metal cations. This selectivity is crucial for biomass purification processes that seek to obtain materials with a high silica content and low metal contamination. Padwal et al. [39] evaluated the influence of DES based on ChCl with different HBDs (oxalic acid (OA), ethylene glycol (EG), and urea) on the extraction of metals present in carbonized rice husk. The results indicated that silicon dioxide (SiO_2_) is the main component of the biomass, accounting for approximately 80.24%, followed by smaller proportions of metal oxides such as K_2_O (4.79%), P_2_O_5_ (2.64%), CaO (0.57%), and MgO (0.89%). These XRF data confirm the high potential of rice husks as a sustainable source of silica, in line with the literature, which highlights the abundance of SiO_2_ in agro-industrial waste.

#### 2.1.4. Scanning Electron Microscopy (SEM)

The morphological characterization of rice husks, both in natura and carbonized, by scanning electron microscopy (SEM) provides essential information on the structural organization of the biomass and the morphological changes induced by heat treatment. SEM images at different magnifications reveal the surface topography, pore distribution, and presence of impurities, all of which may influence the properties and potential applications of the silica extracted from this material [40,41].

**Figure 2:**
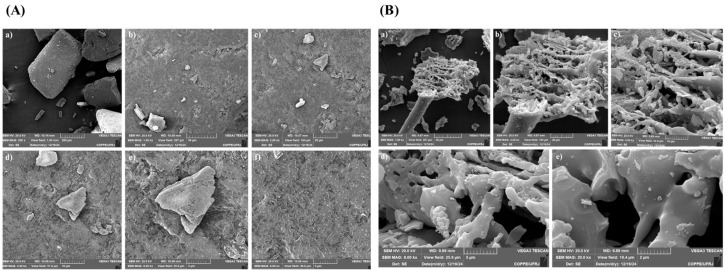
Scanning electron microscopy (SEM) images of in natura rice husk samples at different magnifications. (**A**) Untreated rice husk: (**a**) 200×. (**b**) 1000×. (**c**) 2000×. (**d**) 4000×. (**e**) 8000×. and (**f**) 10,000×. (**B**) Rice husk treated with betaine: lactic acid (1:2) DES: (**a**) 200×. (**b**) 1000×. (**c**) 2000×. (**d**) 4000×. (**e**) 10,000×.

The images of in natura rice husks, shown in Figure 2A, reveal a fibrous and highly structured morphology, typical of the lignocellulosic composition of this material. At magnifications of 200× to 1000×, a relatively compact and organized surface can be seen with interconnected layers that act as a protective barrier for the rice grains. The SEM micrographs reveal a heterogeneous lignocellulosic matrix, while XRF and XRD results indicate that a silica-rich inorganic phase is embedded in this structure, in agreement with previous studies on rice husk composition [39,40,41]. As the magnification increases to 2000× and 4000×, the presence of microcavities and porous regions becomes evident, indicating that the silica is not evenly distributed, but rather deposited irregularly in the structure of the husk. This behavior is expected, as the rice husk acts as a natural biosilicate, accumulating silica from the soil during plant growth. At 8000×, it can be seen that some areas of the surface show agglomerated silica particles, while others still contain residues of organic material, which can affect the efficiency of extracting compounds in industrial processes. The heterogeneity of the surface and the presence of metallic impurities indicate the need for additional chemical treatments to selectively remove these components and improve the purity of the extracted silica.

It is important to highlight that betaine: lactic acid DES was selected for morphological and structural analyses due to its higher removal of the organic fraction, as indicated by the lower residual mass in the thermal analyses and the relative increase in silica content in the carbonized samples. Furthermore, SEM micrographs showed a more homogeneous and porous surface, favoring the characterization of the morphology of the silica exposed after treatment.

Figure 2B shows the analysis of in natura rice husks treated with DES betaine: lactic acid. which reveals changes in the surface morphology of the material. Compared to the untreated sample, there is a partial removal of the outer layer of the husk, which may be associated with the degradation of lignin and other organic components. This modification can be attributed to the combined action of DES in the degradation of lignin and hemicellulose, promoting greater exposure of pores and facilitating adsorption processes and increased efficiency in the removal of metallic impurities. DES are known for their ability to act as efficient extractive agents due to their unique properties, including strong interaction by hydrogen bonds and high solubility for organic and inorganic compounds.

Padwal et al. [39] highlighted that, compared to strong inorganic acids such as HCl, DES offer a more environmentally friendly, efficient and selective approach to the removal of impurities without compromising the structural integrity of the biomass. The results showed that the choice of DES directly influenced the efficiency of metal extraction, with emphasis on the use of systems based on choline-chloride and different hydrogen bond donors (HBD), such as oxalic acid and urea. These solvents demonstrated a superior capacity for removing metal impurities such as Mg, Ca, and Mn, without causing substantial losses in the concentration of silicon in the rice husk.

Similar results were found in this study with the treatment of raw rice husks with DES betaine: lactic acid, resulting in a substantial reduction in the concentration of metallic impurities such as Fe_2_O_3_ and K_2_O, while increasing the relative proportion of the silica fraction in the biomass. This trend suggests that DES act in the selective degradation of organic components such as lignin and hemicellulose, promoting greater accessibility of silica and facilitating its subsequent extraction.

**Figure 3:**
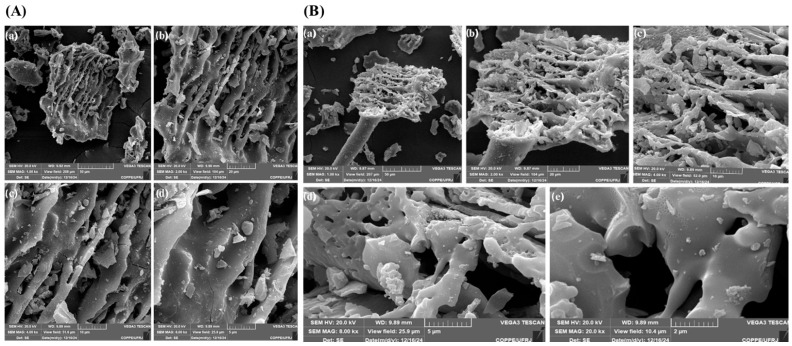
Scanning electron microscopy (SEM) images of carbonized rice husk samples at different magnifications. (**A**) Untreated rice husk: (**a**) 1000×. (**b**) 2000×. (**c**) 4000×. and (**d**) 8000×. (**B**) Rice husk treated with betaine: lactic acid (1:2) DES: (**a**) 1000×. (**b**) 2000×. (**c**) 4000×. (**d**) 8000×. and (**e**) 10,000×.

Figure 3A illustrates the morphology of the rice husk after the carbonization process. At magnifications of 2000× and 4000×, it is possible to see a significant change in the surface structure, with the appearance of cavities and a significant increase in porosity. This phenomenon occurs due to the elimination of the organic fraction of the biomass during pyrolysis, leaving a structure predominantly composed of silica and other residual minerals. In the 8000× images, the carbonized bark shows a highly fragmented morphology, with silica particles dispersed in the carbon matrix. The presence of fissures and interconnected pores suggests an increase in the material’s surface area, potentially making it a candidate for applications as a catalytic support and adsorbent for removing pollutants.

Analysis of the carbonized rice husk treated with betaine: lactic acid DES (Figure 2B) indicates additional structural refinement, possibly due to the removal of remaining impurities. At magnifications of 1000× to 20,000×, a more homogeneous surface and a reduction in the amount of aggregated particles can be seen, indicating that the chemical treatment favors the purification of the silica. In addition, the effects observed after carbonization suggest that the betaine: lactic acid DES treatment influenced the final structure of the carbonized material, reducing the amount of aggregated particles, a higher surface area, a more homogeneous distribution of silica, and increasing the purity of the extracted silica. According to Table 3, there was an increase in the concentration of SiO_2_ in the carbonized bark after treatment with different DES, compared to the untreated sample. This result corroborates the morphological characterization carried out by SEM, which indicated higher porosity and a more homogeneous distribution of silica on the surface of the treated material. Di et al. [42] demonstrated that the treatment of rice husks with DES based on ChCl: oxalic acid-SiO_2_) resulted in modifications to the structural properties of biomass. In addition, the functionalization of silica with DES promotes the development of highly porous and thermally stable surfaces, desirable characteristics for obtaining materials with high purity and chemical reactivity.

#### 2.1.5. X-Ray Diffraction (XRD)

**Figure 4:**
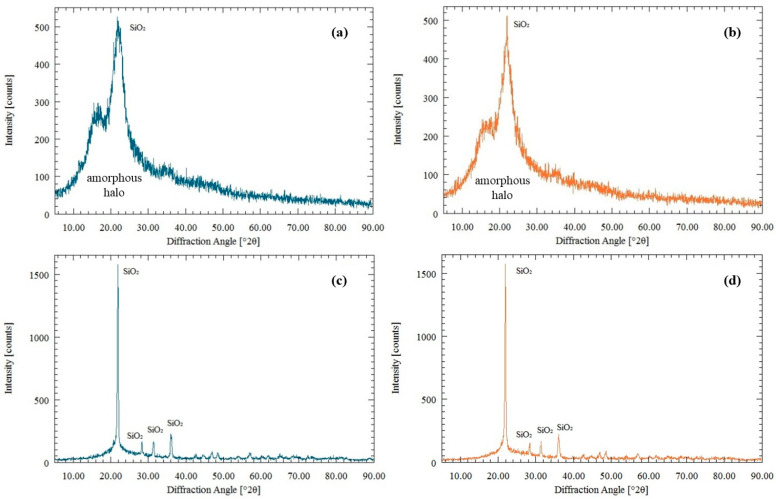
X-ray diffractograms (XRD) of rice husk samples before and after treatment with betaine: lactic acid (1:2) DES at 110 °C. In each panel, the blue curve represents the untreated sample and the orange curve the DES-treated sample: (**a**) in natura rice husk; (**b**) in natura rice husk after DES treatment; (**c**) carbonized rice husk; and (**d**) carbonized rice husk after DES treatment.

Silica can occur either in an amorphous form, which is generally more reactive due to its higher specific surface area and larger number of silanol groups, or in crystalline forms, which are comparatively more inert under typical processing conditions. The relative proportion between these forms depends strongly on the processing method and the temperature applied to the rice husk [43]. For in natura rice husks, an amorphous halo is observed in the 2θ range between 15° and 30°, as shown in Figure 4a. This behaviour is characteristic of predominantly amorphous materials, indicating that the silica present in the raw husk has not undergone significant crystallization. This morphology is maintained due to the mild heat treatments used in the extraction processes. Figure 4b shows the diffractogram of in natura rice husks treated with betaine: lactic acid DES. The treated sample shows a more pronounced amorphous halo, which can be attributed to the combined contribution of the amorphous silica-rich inorganic fraction and residual amorphous organic polymers (cellulose, hemicellulose, and lignin) that are not fully removed by the DES treatment. This observation, together with the lignin and sugar dissolution results, indicates that the DES preserves the amorphous character of the silica while only partially extracting the organic matrix [44]. This behavior is desirable for applications requiring high surface reactivity. Nguyen et al. [8] also observed that silica extracted by conventional chemical treatment predominantly has an amorphous structure, similar to the results observed in the DES-treated samples in this study. The investigation by Di et al. [42] reinforces this approach by demonstrating that DES and silica can be used to optimize biomass conversion processes, promoting greater thermal stability and selectivity in obtaining value-added products. The incorporation of DES into silica improves the dispersion of reagents and favors more efficient catalytic reactions. In the context of extracting silica from rice husks, this type of approach can result in a material with a larger surface area and greater efficiency in removing impurities. consolidating the application of DES in optimizing the quality of the final material.

Figure 4c shows the XRD diffractogram of carbonized rice husk. The peaks at angles of 2θ, 21° and 36° are characteristic of the high-temperature polymorphic phase of silica, known as cristobalite. This result indicates that carbonization at high temperatures promotes the crystallization of amorphous silica into a less reactive phase. As the XRF analysis showed, rice husks contain potassium. This element accelerates the fusion of particles and the crystallization of amorphous silica into cristobalite, as it lowers the melting point of the material [45]. This effect is relevant because controlling the potassium content can directly influence whether amorphous or crystalline silica is obtained during the thermal processes. The influence of the betaine: lactic acid DES treatment can also be seen in Figure 4d. A reduction in the intensity of the crystalline peaks for the treated sample is observed, indicating a higher proportion of amorphous silica. This effect is consistent with studies showing that the use of DES contributes to the removal of metallic impurities, which can catalyze the silica’s crystallization [10,46]. Padwal et al. [39] studied the effect of DES based on ChCl: OA (1:2), ChCl: EG (1:1), and ChCl: urea (1:1) on rice husk. The results showed that the XRD patterns revealed peaks characteristic of crystalline silicon, indicating the preservation of the crystalline structure of Si after treatment. Furthermore, a broadband was observed between 20° and 26°, attributed to the presence of amorphous carbon in the samples.

### 2.2. Lignin Dissolution

**Figure 5:**
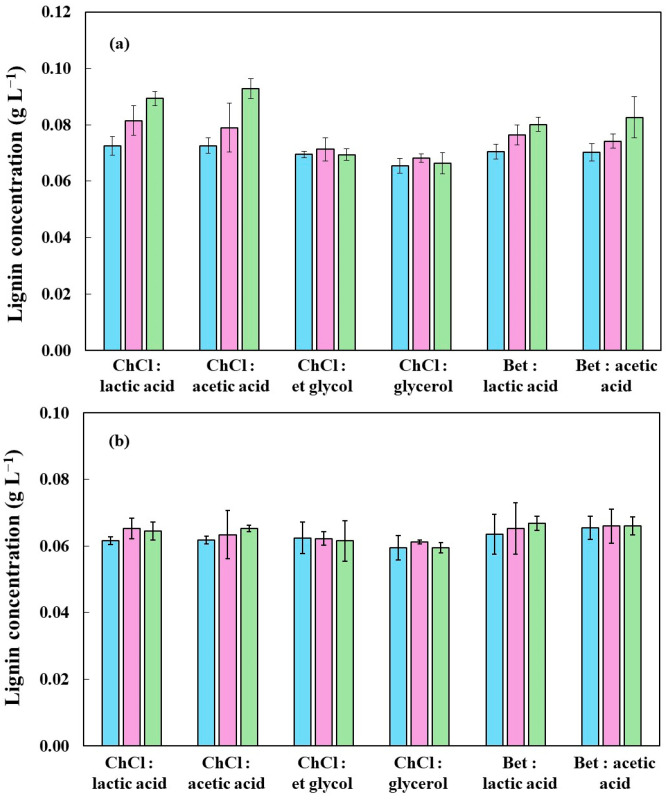
Temperature effect on the lignin dissolution yield of rice husk using deep eutectic solvents: (**a**) in natura and (**b**) carbonized; (■) 110 °C, (■) 130 °C, and (■) 150 °C.

Figure 5 displays the lignin concentration in solution after dissolution of rice husk biomass with various DES at three different temperatures: 110 °C, 130 °C, and 150 °C. The results are presented for both raw (a) and carbonized (b) rice husk, enabling an assessment of how both temperature and biomass influence lignin solubilization.

In general, for all solvents tested, higher temperatures consistently promote greater lignin dissolution, with the most pronounced increases observed between 110 °C and 150 °C. This behavior can be attributed to two primary factors: (i) increasing temperature reduces the viscosity of DES, facilitating its penetration into the lignocellulosic matrix; and (ii) higher thermal energy enhances the cleavage of lignin’s ester and ether bonds, leading to improved solubilization [47,48]. In in natura rice husk, which retains more native lignin, lignin dissolution was significantly higher than in carbonized biomass across all conditions. This reflects the partial degradation and condensation of lignin during pyrolysis, which renders it less accessible to solvent interaction, enhancing the accessibility of cellulose and hemicellulose, enabling the recovery of high-value-added compounds from rice husk biomass [49,50,51]. This behavior is in agreement with studies by Su et al. [52] and Yu et al. [47], which reported reduced delignification in thermally altered lignocellulosic structures due to pseudo-lignin formation and increased recalcitrance.

Among the DES systems evaluated, ChCl: lactic acid and betaine: lactic acid exhibited the highest lignin solubilization yields at all temperatures, particularly at 150 °C. The superior performance of these systems can be linked to the moderate acidity and strong hydrogen bond donor ability of lactic acid, which enhances the disruption of lignin’s complex macromolecular network. Conversely, DES containing glycerol and ethylene glycol as HBDs demonstrated lower delignification efficiency, likely due to their reduced capacity to cleave covalent bonds within the lignin polymer. Interestingly, while ChCl-based DES performed better in carbonized samples, betaine-based DES showed competitive or superior efficiency in raw husk, suggesting that this solvent’s effectiveness is biomass-dependent. Factors such as lignin condensation degree, accessibility of aromatic subunits, and residual hemicellulose content may modulate solvent interaction. The role of temperature in modulating DES performance has also been highlighted by Mamilla et al. [53], who demonstrated that elevated temperatures improve mass transfer kinetics and surface reactivity, consistent with Arrhenius’ law. Additionally, the possible water trace in DES formulations can modulate solvation dynamics, influencing lignin extraction. As observed by Hammond et al. [54] and Buarque et al. [55], low water content (<5 wt%) may enhance hydrogen bonding within DES, lowering viscosity and improving solubilization. However, excess water disrupts the eutectic structure, reducing extraction efficiency.

### 2.3. Total Sugar Content Quantification

**Figure 6:**
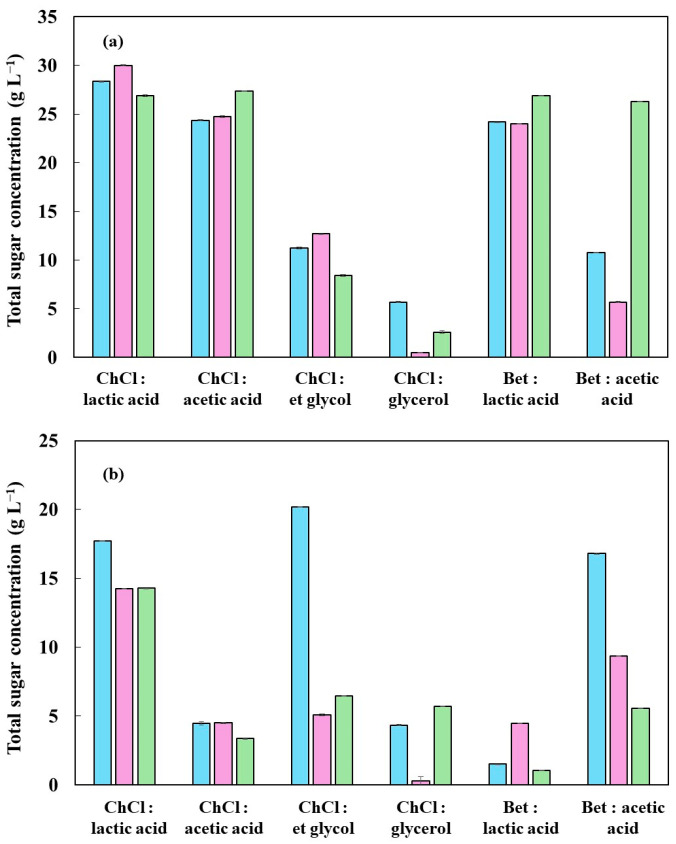
Temperature effect on the total sugar yield of rice husk using deep eutectic solvents: (**a**) in natura and (**b**) carbonized; (■) 110 °C, (■) 130 °C, and (■) 150 °C.

From an analysis of Figure 6, it can be seen that the extraction of total sugars was more efficient with the higher acidity solvents, notably the ChCl: acetic acid, ChCl: lactic acid, betaine: lactic acid, and betaine: acetic acid DES. The remarkable results with acidic DES are associated with their ability to promote the cleavage of glycosidic bonds and to solubilize the polysaccharides in lignocellulosic biomass [56]. Unlike the behavior observed in lignin extraction, the influence of temperature on sugar extraction was not linear. Higher temperatures (130–150 °C) did not result in proportional increases in extraction yield. On the contrary, some DES showed a reduction in yield after 130 °C, possibly due to the thermal degradation of solubilized sugars, such as the formation of furfural and 5-HMF in acidic media, which explains the selection of 110 °C as the ideal condition, as it balances extraction efficiency and the stability of the products obtained [57,58].

In general, the highest sugar extraction yields were obtained from fresh biomass, regardless of the type of DES used. This behavior is to be expected, since fresh biomass preserves its original lignocellulosic structure, containing higher proportions of polysaccharides, especially cellulose and hemicellulose. These fractions are more susceptible to solubilization when exposed to acidic solvents. The presence of accessible glycosidic bonds with a lower degree of condensation favors the hydrolytic action of DES, especially at moderate temperatures (110–130 °C). Carbonized biomass exhibited considerably lower extraction yields, which can be attributed to the partial thermal degradation of carbohydrates during carbonization. The hemicellulose fraction and part of the cellulose are pyrolyzed at high temperatures (>200 °C), forming condensed aromatic compounds and recalcitrant structures such as pseudo-lignin. These residues are less soluble in DES and hinder the release of structural sugars [59].

Another factor to consider is that although carbonization facilitates the removal of the volatile fraction and reduces the total mass of the biomass, it also reduces the organic fraction available for extraction, especially the carbohydrates. Despite its lower efficiency in extracting sugars, carbonized biomass can have advantages in other stages of the valorization process, such as silica purification. Therefore, the choice between using in natura or carbonized biomass must take into account the fractionation objectives, balancing carbohydrate extraction yield with the quality and accessibility of the inorganic matrix.

**Figure 7:**
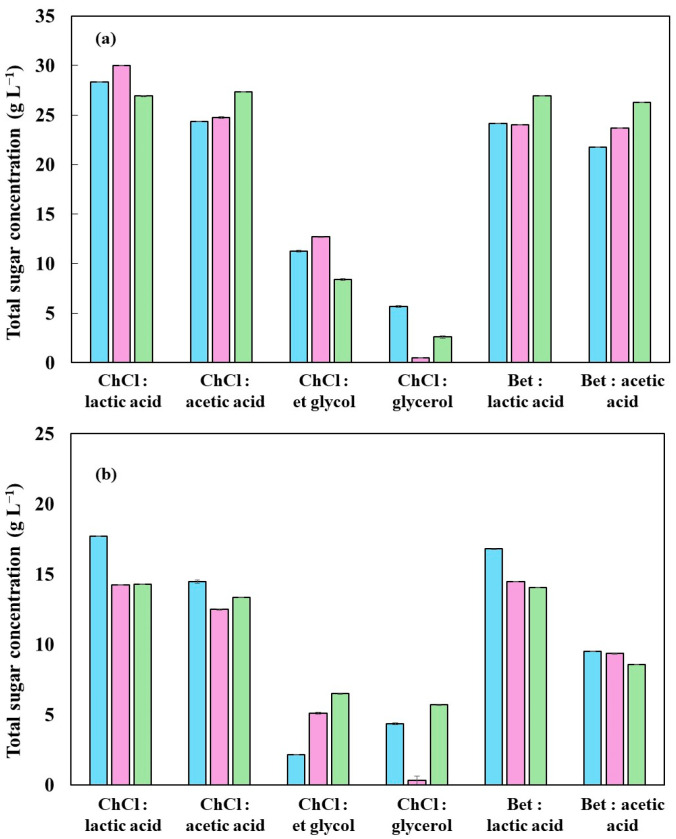
Temperature effect on the glucose concentration of rice husk using deep eutectic solvents: (**a**) in natura and (**b**) carbonized; (■) 110 °C, (■) 130 °C, and (■) 150 °C.

Figure 7 displays the glucose extraction results, by enzymatic hydrolysis, from rice husk samples previously treated with different DES, under three thermal conditions (110 °C, 130 °C, and 150 °C), for (a) in natura and (b) carbonized biomass. In order to validate the results obtained in the total sugar quantification, monomeric glucose was analyzed by HPLC. For this purpose, the residual sample from dissolving the biomass was subjected to enzymatic hydrolysis with the commercial cellulolytic cocktail Cellic CTec2 (Novozymes, containing cellulases, β-glucosidases, and hemicellulases), and the released glucose was quantified. This approach enables a complementary assessment of the treatment’s efficiency, where the lower the amount of glucose produced, the higher the solvent’s efficiency in previously extracting the polysaccharides from the biomass. This behavior is due to the action of DES, which reduces the fraction of structural carbohydrates available for conversion, directly reflecting the low levels of glucose detected.

The results indicate that, in both biomasses, the lowest levels of enzymatically extracted glucose were observed in the samples treated with the acidic DES. This data confirms the high efficiency of these DES in solubilizing structural carbohydrates, reinforcing the findings obtained in the analyses of total sugars (Figure 6). It is noteworthy that the betaine: lactic acid solvents presented the best overall performance, which highlights its favorable combination of acidity, ability to interact with biomass, and extraction selectivity. The tendency for glucose release to increase after 130 °C in some DES may indicate partial thermal degradation of the polysaccharides or structural rearrangements that hinder enzymatic action, an effect already reported in the literature in processes under acidic and high-temperature conditions.

To complement the previous analysis, glucan and xylan were quantified in order to determine the remaining cellulose and hemicellulose content in the biomass treated in natura. The data shown in Table 4 details the composition of the residual biomass after hydrolysis of the material previously treated with DES betaine: lactic acid. The biomass displayed high levels of glucan (26.05%) and xylan (13.96%), indicating the considerable presence of cellulose and hemicellulose. It is important to note that the cellulose and hemicellulose contents correspond to the glucan and xylan fractions, respectively. These results confirm that thermal pre-treatment, combined with the use of DES, results in biomass with a lower content of structural carbohydrates, a highly satisfactory effect when the aim is to purify the mineral fraction (biosilica).

## 3. Materials and Methods

### 3.1. Materials

The rice husk was obtained from Projeto Raiz, located in Porto Alegre, RS, Brazil. Cellic CTec2 was purchased from Novozymes. All the HBA (choline chloride (ChCl) and betaine) and HBD (acetic acid, lactic acid, ethylene glycol, and glycerol) used in the DES formation were purchased from Sigma-Aldrich with purity ≥ 99 wt%. The solvents sulfuric acid, acetone, petroleum ether, phenol, and 1,4-dioxane were purchased from Isofar with purity ≥ 99 wt%.

### 3.2. Preparation of DES

DES were prepared by combining the hydrogen bond acceptor (HBA; choline chloride or betaine) and the hydrogen bond donor (HBD; glycerol, ethylene glycol, lactic acid or acetic acid) in an HBA:HBD molar ratio of 1:2. The mixtures were weighed gravimetrically, placed in sealed vials, and heated in a heat block at 80 °C for 1 h with continuous stirring to ensure homogeneity [60].

### 3.3. Dissolution Process and Biomass Characterization

The raw and carbonized rice husks were milled and sieved using a 32-mesh screen to ensure uniform particle size. The resulting biomass was then characterized by its protein, lipid, ash, and moisture contents using standardized physicochemical methods [61].

TGA Shimadzu (Kyoto, Japan) was also performed to characterize the biomass, following the previously described methodology [62]. The XRF analysis was performed on Rigaku equipment, Miniflex II model, and elemental coal analysis was performed on PerkinElmer equipment, model 2400 Series II CHNS/O Analyzer. XRD analysis of the pastes was taken using a Shimadzu diffractometer (Kyoto, Japan) with a CuKα radiation filter (λ = 1.54059 Å) and operating at 30 kV and 10 mA. Scans were performed with two theta ranges of 5–90 °C with a step increment of 0.05 °C s^−1^. The morphological and crystallographic particle characteristics were investigated using scanning electron microscopy (SEM, Hitachi TM3000 operating at 15 kV, Hitachi High-Technologies Corporation, Tokyo, Japan). Afterwards, DES were added to raw and carbonized rice husks in a biomass-to-solvent ratio of 1:10 (m/m). Then, the samples were inserted into the dry bath without agitation at temperatures of 110, 130, and 150 °C for 1 h. Finally, the samples were centrifuged, the supernatant was separated, and the dissolution yield was determined using Equation (2).

(2) $$\%\;Dissolution=\frac{C_{i}\;\times \;V}{m_{t_{i}}}$$

where $C_{i}$ is the biomass concentration in mg mL^−1^, *V* is the system volume in mL, $m_{t_{i}}$ is the total mass of the lignin.

### 3.4. Lignin Determination

Lignin content was quantified following the method proposed by Ibrahim et al. [63]. The lignin residue was solubilized in a 90 wt% aqueous dioxane solution. All analyses were performed in triplicate to ensure accuracy and reproducibility. The lignin dissolution process was evaluated using the alkaline lignin standard (Aldrich cod. 370959, lot#MKBJ0452V).

### 3.5. Total Sugar Determination

Total sugar content was determined using the phenol-sulfuric acid method described by Dubois et al. [64]. In this procedure, 10 mg of the supernatant was mixed with 270 μL of distilled water, 270 μL of 5 wt% phenol solution, and 1460 μL of 98 wt% sulfuric acid. The mixture was allowed to stand for 30 min at room temperature to ensure complete reaction. Absorbance measurements were recorded at 490 nm using a spectrophotometer to quantify total sugar content.

### 3.6. Determination of Sugars Using HPLC

According to the supplier, the enzyme Cellic CTec2 from Novozymes contains cellulases, beta-glycosidases, and hemicellulases in its composition. Therefore, the enzymatic hydrolysis of the residual biomass was performed following the protocol described by Pin et al. [65]. In the reaction, 10 mg of substrate was mixed with 0.9 mL of 50 mM citrate buffer (pH 5) and 100 μL of Cellic CTec2 enzyme. The reaction was conducted at 50 °C with 300 rpm agitation for 48 h using a Thermomixer Eppendorf. After incubation, the samples were centrifuged and filtered through 0.22 μm membrane filters before being analyzed by HPLC (Shimadzu, Kyoto, Japan). Chromatographic analysis was carried out using an Aminex HPX-87H column (BioRad, 300 × 7.8 mm), with detection performed using a RID-10A refractive index detector and a LC-20ADSP binary pump. The mobile phase consisted of a 5 mM H_2_SO_4_ aqueous solution, with a flow rate of 0.6 mL min^−1^, an oven temperature of 55 °C, and an injection volume of 20 μL, using glucose as the standard.

## 4. Conclusions

This study demonstrated the feasibility of using DES as sustainable and selective media for rice husk fractionation. Acidic DES, particularly those based on lactic acid, achieved high efficiency in removing lignin (>70%) and solubilizing structural sugars, while simultaneously increasing the purity of the inorganic fraction. XRF analyses confirmed that the betaine: lactic acid DES promoted biosilica with SiO_2_ content above 80%, a significant improvement compared to untreated carbonized husks (76.36%). Furthermore, TGA, SEM, and XRD results showed that DES treatment enhanced porosity and surface homogeneity, while preserving the amorphous structure of silica, an essential characteristic for high-reactivity applications. The controlled acidity of DES proved decisive in cleaving ester and glycosidic bonds in the lignocellulosic matrix without compromising silica integrity. The optimized condition of 110 °C balanced extraction efficiency with product stability, minimizing sugar degradation. This work highlights DES as a versatile, tunable, and environmentally compatible platforms that surpass conventional thermal and chemical methods by reducing impurities and improving silica quality. In addition to advancing the sustainable valorization of agricultural residues, it also establishes DES as a promising route for producing high-purity biosilica within the framework of green chemistry and the circular bioeconomy.

## Figures and Tables

**Table 1:** Physico-chemical characterization of in natura and carbonized rice husks.

Rice Husks	In Natura (%)	Carbonized (%)
Lipids	0.79 ± 0.25	0.79 ± 0.52
Proteins	1.95 ± 0.12	0.81 ± 0.01
Ash	18.61 ± 0.46	91.36 ± 0.06
Moisture	7.52 ± 0.08	1.86 ± 0.01
Carbohydrates	71.13 ± 0.08	5.18 ± 0.01

**Table 2:** Total mass loss (%) for in natura and carbonized rice husks after treatment with different DES systems at 110 °C. Values correspond to the overall mass loss measured by the TGA thermograms.

DES	Total Mass Loss—In Natura (%)	Total Mass Loss—Carbonized (%)
Untreated	59.84	8.19
ChCl: lactic acid	76.89	7.89
ChCl: acetic acid	78.45	8.11
ChCl: ethylene glycol	84.49	7.37
ChCl: glycerol	86.30	8.27
Betaine: lactic acid	83.60	8.14
Betaine: acetic acid	82.58	9.01

**Table 3:** Percentage of chemical content in rice husk obtained from XRF before and after DES treatment.

DES	SiO_2_	K_2_O	CaO	Ag_2_O	Fe_2_O_3_	Al_2_O_3_	P_2_O_5_	LOI *
in natura rice husk								
Untreated	12.97	0.92	0.76	0.92	0.97	0.77	0.30	81.37
ChCl: lactic acid	15.10	0.35	0.77	1.61	0.84	0.80	0.61	76.89
ChCl: acetic acid	16.30	0.35	0.74	1.13	0.89	0.70	0.35	78.45
ChCl: ethylene glycol	11.69	0.27	0.68	0.34	0.81	0.67	0.46	84.49
ChCl: glycerol	11.80	0.21	0.38	0.16	0.43	0.32	-	86.30
Betaine: lactic acid	12.37	0.32	0.67	-	0.87	0.77	0.38	83.60
Betaine: acetic acid	13.06	0.43	0.69	-	0.88	0.79	0.45	82.58
carbonized rice husk								
Untreated	76.36	5.52	2.26	0.91	2.10	1.66	1.46	8.85
ChCl: lactic acid	79.91	3.59	2.40	0.87	1.82	1.44	1.19	7.89
ChCl: acetic acid	77.15	3.41	2.21	1.49	2.14	1.49	1.56	8.11
ChCl: ethylene glycol	80.95	4.16	2.10	0.42	1.18	0.89	1.02	7.37
ChCl: glycerol	78.46	4.04	2.17	0.93	1.20	0.79	0.67	8.27
Betaine: lactic acid	80.22	3.43	2.18	0.65	1.38	1.14	0.93	8.14
Betaine: acetic acid	77.90	3.68	2.04	1.64	2.23	1.47	0.67	9.01

* Loss on Ignition corresponds to the fraction of mass lost during the calcination of the sample, associated mainly with the decomposition of organic matter and the release of undetected volatile compounds.

**Table 4:** Compositional analysis of rice husk biomass after treatment with the betaine: lactic acid DES (1:2 molar ratio) by HPLC at 110 °C.

Characterization	In Natura (%)
Extractives	8.11
Lignin	48.16 ± 2.25
Glucan	26.05 ± 0.80
Xilan	13.96 ± 0.47

## Data Availability

Data will be made available on reasonable request.

## Supplementary Materials

The following supporting information can be downloaded at: https://www.mdpi.com/article/10.3390/molecules30244697/s1, Table S1: Water content and pH of DES preparations.; Table S2: Weight loss by temperature (T.—°C) range and decomposition temperature (Tdec—°C) for green solvents based on choline chloride.
