# Supplementary material for: Deep Eutectic Solvents as a Sustainable Approach for Silica Recovery from Rice Husk
Source: Molecules. 2025 Dec 8;30(24):4697. doi: 10.3390/molecules30244697 (PMC12735558; doi:10.3390/molecules30244697)
Supplement: Supplementary file 1 [file molecules-30-04697-s001.zip › molecules-4015471-supplementary.pdf]

Support Information

Deep eutectic solvents as a sustainable approach for silica recovery from rice husk

Célio S. Faria-Júnior, Lucas dos Santos Silva, Armando L. C. Cunha, Filipe S. Buarque*, Bernardo Dias. Ribeiro

School of Chemistry, Federal University of Rio de Janeiro, Av. Athos da Silveira Ramos, 149. Ilha do Fundão, 21941-909, Rio de Janeiro Brazil.

* Correspondence: filipesmith@eq.ufrj.br

1. General methods

DES characterization: The water content of each DES after its preparation was measured in triplicate using Karl Fischer titration (Metrohm 870 KF Titrino Plus). Thermogravimetric Analysis (TGA) was performed to characterize the thermal stability of the solvents. TGA instrument was a Shimadzu Model TGA-50 thermal analyzer, with the samples measured at flow rate of 60 mL min^{-1} and approximately 10 mg of each material was used for the analysis. Moreover, the temperature was ramped from 25 °C to 900 °C at a constant rate of 10 °C min^{-1}.

2. Characterization of DESs

The characterization of solvents is essential to understand their physicochemical properties and thermal stability. These parameters define their efficiency in interacting with biomass and their selectivity in extracting different fractions. This study investigated using DES based on ChCl or betaine with different HBDs (glycerol, ethylene glycol, lactic acid, and acetic acid). Table S1 shows the essential physicochemical characterizations for a better understanding of its interaction with lignocellulosic materials such as rice husks. The water content in DESs ranged from 1.16% to 5.09%, with ChCl: ethylene glycol and ChCl: acetic acid having the highest values. Traces of water (<5%) are common in DESs due to their hygroscopic nature, especially in choline-based systems. Moreover, small amounts of water can act as a cosolvent, reducing viscosity and facilitating the diffusion of the solvent into the lignocellulosic matrix, thereby enhancing the extraction of lignin and sugars. However, excessive content can disturb the eutectic structure of the solvent, decreasing its effectiveness, which explains why levels below 5% should be maintained in order to preserve the integrity of the hydrogen bonding interactions between HBA and HBD [33].

Table S1: Water content and pH of DES preparations.

DES	Water content (%)	pH
ChCl: lactic acid	1.16 ± 0.26	1.31 ± 0.09
ChCl: acetic acid	3.41 ± 0.16	1.12 ± 0.07
ChCl: ethylene glycol	5.09 ± 0.32	5.22 ± 0.10
ChCl: glycerol	1.65 ± 0.17	5.09 ± 0.31
Betaine: lactic acid	1.97 ± 0.08	1.15 ± 0.28
Betaine: acetic acid	2.91 ± 0.11	1.23 ± 0.19

The pH values of the DESs varied considerably, indicating different interaction potentials with the lignocellulosic biomass. In general, DESs composed of carboxylic acids (such as acetic acid and lactic acid) had a significantly more acidic pH. In contrast, solvents containing alcohol, such as

glycerol and ethylene glycol, resulted in a more neutral to slightly acidic pH (pH 5.09 and 5.22, respectively). This acidity plays a central role in the ability of DES to act as an extractive agent. Solvents with a lower pH more strongly promote the cleavage of ester and ether bonds present in the lignin structure, facilitating its solubilization. Furthermore, high acidity contributes to the hydrolysis of glycosidic bonds, promoting the extraction of structural sugars from biomass [34,35].

Table S2 displays the mass loss ranges and decomposition temperatures (T_{dec}). It is observed that ChCl: lactic and ChCl: glycerol-based DESs exhibited higher Tdec values in regions II and III (238.9-301.7 °C and 217.7-299.2 °C, respectively), indicating greater thermal stability. This characteristic is advantageous, since it allows these solvents to be applied in pretreatments at moderate temperatures (110-150 °C, conditions used in this study) without the risk of significant solvent degradation. DES based on acetic acid showed lower initial decomposition temperatures (81.0 and 68.3 °C, respectively), which gives them lower thermal stability. This fragility, however, is associated with the high acidity of these solvents (see Table 1), which, as mentioned in the study, promotes the cleavage of ester and glycosidic bonds in biomass, leading to higher lignin and sugar extraction. Another relevant point is the performance of betaine: lactic acid, with a wide stability range (125.9-286.7 °C) and T_{dec} around 239 °C. This profile indicates an attractive combination of moderate acidity and thermal resistance.

Table S2: Weight loss by temperature (T. - °C) range and decomposition temperature (T_{dec} - °C) for green solvents based on choline chloride.

**choline-based HBA**	**Region I**		**Region II**		**Region III**	
	**T. range (°C)**	**T_{dec} (°C)**	**T. range (°C)**	**T_{dec} (°C)**	**T. range (°C)**	**T_{dec} (°C)**
ChCl: lactic acid	129.2–198.7	171.2	201.90–250.1	238.87	293.40– 315.4	301.7
ChCl: acetic acid	41.22–174.87	81.0	213.51–268.3	244.19	281.64–318.1	299.4
ChCl: EG	61.69–181.3	144.82	234.59–273.8	251.72	267.57–332.9	308.5
ChCl: glycerol	148.45–258.4	217.7	263.31–321.4	299.19	-	-
Betaine: lactic acid	125.99– 286.7	238.9	-	-	-	-
Betaine: acetic acid	38.63–113.5	68.3	191.52–229.6	211.08	269.72–314.2	303.6
